# Optimal Resource Allocation via Unified Closed-Form Solutions for SWIPT Multi-Hop DF Relay Networks

**DOI:** 10.3390/s26020512

**Published:** 2026-01-12

**Authors:** Yang Yu, Xiaoqing Tang, Guihui Xie

**Affiliations:** 1 Electronic Information School, Hubei Three Gorges Polytechnic, Yichang 443199, China; 2 School of Artificial Intelligence, Hubei University, Wuhan 430062, China; 2010202120076@whu.edu.cn; 3 School of Automation, China University of Geosciences (Wuhan), Wuhan 430074, China; xieguihui@cug.edu.cn

**Keywords:** convex optimization, closed-form solution, decode-and-forward, multi-hop relay, power splitting, system throughput maximization, source power minimization, simultaneous wireless information and power transfer

## Abstract

Multi-hop relaying can solve the problems of limited single-hop wireless communication distance, poor signal quality, or the inability to communicate directly by “relaying” data transmission through multiple intermediate nodes. It serves as the cornerstone for building large-scale, highly reliable, and self-adapting wireless networks, especially for the Internet of Things (IoT) and future 6G. This paper focuses on a decode-and-forward (DF) multi-hop relay network that employs simultaneous wireless information and power transfer (SWIPT) technology, with relays operating in a passive state. We first investigate the optimization of the power splitting (PS) ratio at each relay, given the source node transmit power, to maximize end-to-end network throughput. Subsequently, we jointly optimized the PS ratios and the source transmit power to minimize the source transmit power while satisfying the system’s minimum quality of service (QoS) requirement. Although both problems are non-convex, they can be reformulated as convex optimization problems. Closed-form optimal solutions are then derived based on the Karush–Kuhn–Tucker (KKT) conditions and a recursive method, respectively. Moreover, we find that the closed-form optimal solutions for the PS ratios corresponding to the two problems are identical. Through simulations, we validate that the performance of the two proposed schemes based on the closed-form solutions is optimal, while also demonstrating their extremely fast algorithm execution speeds, thereby proving the deployment value of the two proposed schemes in practical communication scenarios.

## 1. Introduction

### 1.1. Background

Multi-hop relaying is a key technology in wireless communication networks. Its core concept is to establish communication links between senders and receivers by forwarding data through one or more intermediate nodes, i.e., relay nodes. Multi-hop relaying technology achieves data transmission by decomposing a long-distance, low-quality communication link into multiple short-distance, high-quality links. The relay nodes receive signals from the source node or the previous relay node, process them (e.g., through amplification or decoding), and then forward them to the next node or the final destination [1]. This hop-by-hop communication strategy effectively overcomes the limitations of traditional single-hop communication, with its superiority becoming particularly evident in complex and challenging communication environments.

First, multi-hop relaying significantly expands communication coverage. When base station signal coverage is limited, or there are severe shadowed areas (such as buildings or terrain obstacles), multi-hop relaying can effectively extend the network coverage range, enabling users at the cellular edge or farther distances to access the network [2].

Secondly, multi-hop relaying contributes to throughput enhancement, particularly in cellular network edge areas. By establishing more reliable short-distance links, multi-hop relaying can improve data transmission rates and overall network throughput [3].

Thirdly, multi-hop relaying also helps improve energy efficiency. In wireless sensor networks (WSNs), nodes are typically battery-powered, making energy a limited resource. Multi-hop routing protocols are designed to optimize energy consumption by selecting paths with lower energy usage, thereby prolonging network lifetime [4]. Some studies have proposed energy-efficient sensing and transmission schemes that maximize energy efficiency through joint optimization of sensing time and power allocation [5].

Finally, by providing multiple paths and link selection mechanisms, multi-hop relaying enhances communication reliability, particularly in environments with transient link interruptions or adverse channel conditions. For instance, in free space optical (FSO) communications, multi-hop relay transmission can significantly improve link performance and reliability, despite FSO links being highly susceptible to atmospheric effects [6].

Building on these advantages, along with its notable network configuration flexibility, multi-hop relaying systems are widely applicable in Internet of Things (IoT), WSNs, and emergency communication networks, serving as a key solution for these network architectures. However, in wireless multi-hop relay networks, relay nodes are generally powered by built-in batteries. In such cases, when the battery power is depleted, manual replacement or recharging becomes necessary. This results in substantial labor costs across scenarios such as large-scale IoT deployments, industrial monitoring systems, and remote areas [7]. On the other hand, battery lifespan is not fixed but is influenced by factors such as ambient temperature, depth of discharge, and the number of charge–discharge cycles, which further adds complexity to network maintenance.

Simultaneous wireless information and power transfer (SWIPT) is an emerging wireless communication paradigm that allows receiving devices to simultaneously acquire information and harvest energy from the same radio frequency (RF) signal [8]. The core concept of this technology leverages the physical nature of radio waves, which carry both information and energy during transmission, thereby enabling wireless power delivery to energy-constrained devices and resolving the inherent dilemma between energy consumption and information transmission in conventional wireless communication systems.

Currently, the mainstream implementation strategies for SWIPT technology include time switching (TS) and power splitting (PS) [9,10,11]. In the former approach, the receiver performs information decoding (ID) and energy harvesting (EH) in different time slots. This method is straightforward and intuitive, but requires precise time synchronization and scheduling mechanisms. In the latter approach, the receiver allocates the power of the received RF signal proportionally between the information decoder and the energy harvester. This method enables concurrent information transmission and EH but demands efficient power splitters and highly sensitive receiver circuits.

By applying SWIPT technology in multi-hop relay networks, relay nodes can reduce their reliance on conventional batteries, thereby lowering the substantial labor costs associated with frequent battery replacements and mitigating the impact of environmental factors (such as temperature and depth of discharge) on battery lifespan, thus simplifying the complexity of network maintenance. The application of SWIPT in multi-hop relay networks represents an active research area in the field of wireless communications. Numerous studies have focused on effectively integrating EH with information transmission to address challenges posed by energy-constrained relay nodes, particularly in enhancing network lifespan, reducing operational costs, and strengthening network security.

### 1.2. Related Work

A significant portion of research on multi-hop relay networks employing SWIPT technology focuses on two-hop relay scenarios. For instance, Ref. [12] proposes a two-way relay energy accumulation communication system aimed at addressing the issue of neglected energy accumulation and storage in existing SWIPT technologies. This system maximizes the instantaneous transmission rate of the system link and the system equivalent revenue through the optimization of relay selection, time slot allocation factors, power allocation factors, and relay transmit power. Reference [13] considers a dual-hop SWIPT-enabled multi-relay orthogonal frequency division multiplexing (OFDM) system for IoT applications. To reduce computational complexity, a time-sharing strategy and the Lagrangian dual method were employed to reformulate the optimization problem, which was subsequently proven to be convex. Ultimately, the study proposed a joint optimization approach for power and subcarrier allocation to maximize the transmission rate of the dual-hop multi-relay IoT system. The authors of [14] investigated the rate-energy trade-off precoding design in a SWIPT-based two-way relay system. The study employs chordal distance (CD) decomposition for balanced precoder design, which significantly reduces computational complexity compared to conventional convex relaxation schemes and offers flexibility in adapting to dynamic energy demands. Given non-negative CD values, the proposed balanced precoder achieves higher EH amounts than perfect interference alignment (IA) precoders. Reference [15] investigates the outage performance of SWIPT using either static or dynamic PS schemes in a dual-hop decode-and-forward (DF) relay system where a direct link exists between the source and destination nodes. The results demonstrate that the system achieves full diversity gain regardless of whether static or dynamic PS schemes are used, and the dynamic PS scheme outperforms the static scheme. Reference [16] investigates a TS-based cooperative non-orthogonal multiple access (NOMA) network comprising a base station (BS), a near user, a distant user, and a full-duplex relay. The authors derive closed-form expressions for the throughput of both near and distant users under static battery energy and dynamic battery energy schemes, considering a threshold-based nonlinear EH model. Furthermore, for the dynamic battery energy scheme, precise closed-form expressions for the average battery energy consumed per symbol interval are derived. The study reveals that the selection of battery energy and TS parameters is critical to achieving maximum throughput for the distant user while ensuring the target throughput is attained for the near user. In [17], the authors proposed a performance analysis framework for SWIPT-enabled two-way relay networks with hybrid receivers over Nakagami-m fading channels. Based on this framework, analytical expressions were derived for the outage probability (OP), asymptotic OP, system throughput, energy efficiency, and ergodic capacity. The study further examined the effects of TS factors, PS ratios, energy conversion efficiency, nonlinear power amplifier (NLPA), and threshold data rates on the network performance. The authors of [18] investigated the resource allocation and relay selection problem in a dual-hop relay-assisted multi-user OFDMA network, aiming to maximize the system sum rate by optimizing the PS ratios of terminal nodes as well as relay, carrier, and power allocation under EH and transmit power constraints. Due to the complex mixed-integer nonlinear programming structure of the problem, this paper proposes two solution approaches: one solves its dual problem to achieve asymptotic optimality and reduce execution time, while the other is a lower-complexity heuristic method. The study also considers a more practical nonlinear EH model.

Research on multi-hop (three or more hops) relay networks employing SWIPT technology primarily focuses on how to effectively coordinate information transmission and EH among multiple relay nodes to optimize overall network performance. The complexity of such studies is significantly higher compared to dual-hop networks, as they must account for additional relay stages, cumulative signal attenuation, as well as energy management and resource allocation strategies across the relay nodes. For instance, Ref. [19] jointly considers SWIPT and routing selection in multi-hop energy-constrained wireless networks. To reduce energy consumption, the authors first formulated the problem of information and energy allocation for links in forwarding paths and solved it through an iterative algorithm. Furthermore, a novel routing metric was proposed to assess the energy consumption difference between links utilizing SWIPT and those without SWIPT. The authors of [20] investigated a multi-input multi-output (MIMO) amplify-and-forward (AF) multi-hop WSN equipped with SWIPT technology to achieve energy self-sustainability. Under the constraints of transmit power and the EH requirements of the destination node, the authors proposed an alternating optimization algorithm based on diagonalization processing to maximize the achievable transmission rate of the WSN by jointly optimizing the precoding beamforming schemes of the source and relays. Regarding the carried energy and security of relay nodes in EH multi-hop device-to-device (D2D) networks, Ref. [21] investigates a relay selection mechanism in the presence of eavesdroppers, derives an upper bound for the end-to-end secrecy connectivity probability, and proposes a relay selection algorithm to construct relay paths with enhanced security. To mitigate the intermittent on-off behavior of energy harvesters that use supercapacitors instead of batteries, Ref. [22] proposes three strategies for integrating battery-free EH devices into industrial multi-hop WSNs. For each proposed strategy, the authors discuss their applicability by considering multiple critical factors, such as energy source type, storage capacity, device mobility, latency, and reliability. In the multi-hop relay network studied in [23], all nodes are equipped with battery-assisted EH technology to collect energy from power beacons. The authors derived analytical expressions for outage probability, throughput, average battery energy consumption, and battery energy efficiency. The study demonstrates how parameter selection can be optimized to achieve maximum network throughput, minimize average battery energy consumption while meeting target throughput requirements, and maximize overall battery energy efficiency. Reference [24] proposes a hybrid SWIPT architecture that integrates TS and PS schemes based on an EH mechanism utilizing multiple energy sources. Additionally, an efficient routing mechanism incorporating an energy cost metric is introduced to minimize the total energy consumption along the path. Reference [25] adopts a log barrier interior-point method to solve the convex optimization of TS ratios for throughput maximization in multi-hop SWIPT-DF relay networks, demonstrating superior performance and computational efficiency.

Although the above studies have made significant progress in resource allocation for SWIPT-enabled relay networks, they primarily focus on dual-hop scenarios or rely on iterative numerical methods for optimization. There remains a lack of closed-form optimal solutions for multi-hop DF relay networks with passive (battery-free) nodes, which is critical for low-complexity, real-time implementations in practical IoT and sensor networks.

Furthermore, Ding et al. [26] provide a comprehensive overview of smart antenna technologies, such as MIMO and relaying, in SWIPT systems, discussing various receiver architectures (e.g., PS and TS) and their trade-offs in terms of energy efficiency and spectral efficiency. This work complements our study by highlighting the broader context of antenna technologies in multi-node networks, which aligns with our focus on resource allocation for passive multi-hop DF relays.

### 1.3. Motivation, Contribution, and Organization

In this paper, we focus on a DF multi-hop relay network. The DF protocol is chosen over AF primarily to mitigate the detrimental effect of noise accumulation over multiple hops. By decoding and regenerating the information signal at each relay, the DF strategy prevents the amplification and forwarding of noise, which is crucial for maintaining signal integrity in a power-limited SWIPT system where the received signal power at a relay can be low. This makes DF particularly suitable for the considered scenario. Each relay node in the network employs a PS-SWIPT scheme, harvesting energy from a portion of the RF signal received from the preceding node and storing it in a supercapacitor. This harvested energy is then used to forward the decoded information to the next node. Therefore, the relays in the network can operate in a passive state without relying on internal batteries or external power sources.

This network paradigm is particularly suited for practical applications where reliable power grid access is unavailable, or battery replacement is prohibitively costly or dangerous. Specifically, it can be deployed in WSNs transmitting data along specific routing paths [27,28], where energy-constrained sensor nodes benefit from passive relaying to achieve energy self-sustainability and extend network lifetime without battery maintenance. Similarly, in industrial monitoring systems [29], such as within a factory or warehouse environment, sensors and actuators need to communicate across large areas but are often constrained to short-range, low-power transmissions per hop to minimize interference and comply with safety regulations. Furthermore, in post-disaster emergency communication systems [30], the rapid deployment of a reliable communication backbone is critical, and relays can be placed at strategic, short-distance intervals to ensure connectivity without relying on pre-existing power infrastructure. While cascade fading effects pose a challenge in any multi-hop system, these targeted scenarios often involve controlled environments or line-of-sight (LoS) conditions where individual hop distances are intentionally kept short, thereby mitigating severe path loss and cumulative signal degradation. The primary value proposition of our proposed passive relay network in these applications is the significant reduction in long-term maintenance effort and cost achieved by eliminating the need for battery replacements, a benefit that persists even when accounting for the inherent inefficiencies of multi-hop energy transfer.

It is well-recognized that equipping relay nodes with multiple antennas (MIMO) can further improve the efficiency of wireless power and information transfer through advanced beamforming techniques [20]. However, the primary focus of this paper is on the fundamental and widely applicable scenario where each node is equipped with a single antenna. This allows us to derive tractable, closed-form optimal solutions that provide key insights and serve as a performance benchmark. The extension of the proposed framework to multi-antenna relay systems is a promising direction for future work, as discussed in the conclusion.

In contrast to the battery-equipped multi-hop relay network in [23], which focuses on maximizing network throughput, minimizing average battery energy consumption, and maximizing overall battery energy efficiency, this paper addresses the underexplored niche of passive (battery-free) multi-hop DF relay networks, where relays are solely powered by SWIPT. This paradigm eliminates the need for battery maintenance and is particularly suitable for scenarios where reliable grid power is unavailable. We investigate the optimization of PS ratio allocation in a multi-hop passive relay network to achieve two objectives: maximizing system throughput under a given source node transmit power, and minimizing source node transmit power while satisfying a minimum quality of service (QoS) requirement. The innovations and contributions of this paper are as follows:

1. First, this paper aims to solve for the optimal PS ratio at each relay that maximizes the system throughput of a multi-hop passive relay network under a given source node transmit power. We transform the non-convex throughput maximization problem into a convex optimization form and derive closed-form solutions for the PS ratio of each relay node based on the KKT conditions and a recursive method. By solving this problem, the end-to-end transmission quality of information routing can be effectively optimized in low-complexity IoT systems that support our proposed passive relay network paradigm and operate with a constant transmit power at the source node.
2. Building upon the solution for maximizing system throughput under a given source node transmit power, this paper further investigates the source power minimization (SPM) while satisfying a minimum QoS requirement by jointly optimizing the source node transmit power and the PS ratios of relay nodes. Addressing this problem helps alleviate the burden on source node power resources during information routing in IoT networks. The optimal solution corresponding to this problem is also derived based on the KKT conditions and a recursive method, and the closed-form solutions for the PS ratios are identical to those obtained for the system throughput maximization (STM)problem.
3. The simulation section of this paper verifies the optimality of the two proposed schemes. The advantages of applying the proposed system model and schemes to sensor networks are elucidated: the low computational complexity of the algorithms and the ability of the second scheme to extend the network’s operational lifetime. The proposed schemes are compared with fixed PS ratio schemes in simulations, and the results demonstrate that the proposed schemes significantly outperform the benchmark.

To further clarify the significant novelty of this work within the well-established field of SWIPT, Table 1 provides a comparative summary between this paper and several representative studies from the literature. The key distinctions of our work lie in (1) the focus on a multi-hop network with passive (battery-free) DF relays powered solely by SWIPT; (2) the joint investigation of both the STM problem under a fixed source power and the SPM problem under a QoS constraint; and (3) most importantly, the derivation of unified closed-form optimal solutions for the underlying non-convex problems, which enables extremely fast and optimal resource allocation compared to iterative numerical approaches commonly employed in the literature [12,13,19,20].

The structure of the remainder of this paper is organized as follows: Section 2 presents the system model of a multi-hop DF relay network employing PS-based SWIPT technology, enabling relay nodes to operate in a passive state. Subsequently, two optimization problems are sequentially proposed. The first aims to maximize the system throughput by allocating the PS ratios of the relay nodes under a given source node transmit power. The second aims to minimize the transmit power of the source node while satisfying the minimum QoS requirement of the system. In Section 3, we first equivalently reformulate these two non-convex optimization problems into convex optimization problems. Then, based on the KKT conditions and a recursive approach, we derive their closed-form optimal solutions. Furthermore, we discuss the implementation of the proposed resource allocation schemes based on these closed-form solutions in real-world scenarios. In Section 4, simulations validate the optimality of the two proposed schemes. Their performance is evaluated through comparisons with benchmark algorithms, demonstrating their potential in practical communication scenarios. Finally, Section 5 concludes the paper.

## 2. System Model and Problem Formulation

**Figure 1:**
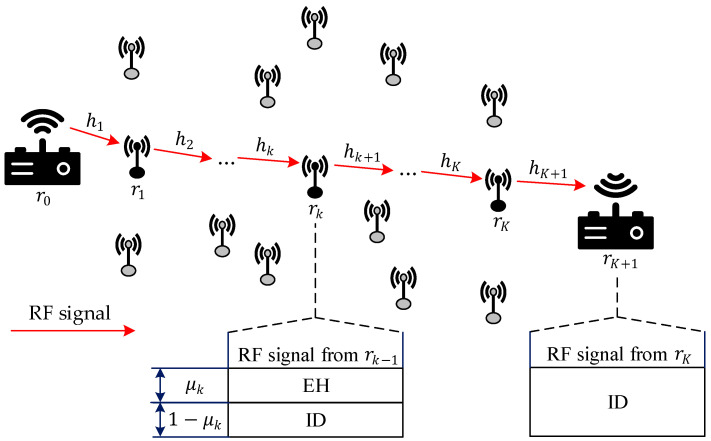
Multi-hop DF relay communication network employing PS-SWIPT.

Table 2 lists the main symbols used in this paper. In this section, we first introduce the considered multi-hop relaying communication network model, as shown in Figure 1. In this network, we denote the source node as $r_{0}$, which forwards information hop-by-hop through the relays in the set $\left\{r_{1},\ldots r_{K}\right\}$ to the destination node $r_{K+1}$. It is assumed that the source node is powered by an internal battery or an external power supply, while all the relays operate in a passive state. Specifically, relay $r_{k}$, k∈{1,…,K}, employs the SWIPT technology to harvest energy from the RF signals of its previous-hop neighboring node. Using the PS protocol, it simultaneously decodes and forwards data packets to the next-hop neighboring node. Denoting the PS ratio as $\mu_{k}\in \left(0,1\right)$ (Practical power splitters have finite resolution and dynamic range constraints. Although we assume ideal PS ratios in (0,1), our closed-form solutions, e.g., Equation (36), can be adapted to discrete values in real devices), the RF signal at relay $r_{k}$ is divided into two streams: a portion, $\mu_{k}$, is used for EH, and the remaining portion $1-\mu_{k}$ is used for ID. Furthermore, we assume that all nodes are equipped with a single antenna and operate in half-duplex mode, meaning that a relay node forwards data only after completing the reception of information. The channel coefficient between nodes $r_{k-1}$ and $r_{k}$ is denoted as $h_{k}$, k∈{1,…,K+1}, and it remains constant during the time period (transmission frame) when data is transmitted from one node to the next adjacent node.

In this paper, the channel coefficients $h_{k}$ for each hop are assumed to be independent and identically distributed (i.i.d.) to ensure analytical tractability and facilitate the derivation of closed-form solutions. This assumption is common in foundational studies of multi-hop SWIPT systems [23,25] as it decouples the per-hop resource allocation problems. However, we acknowledge that in practical deployments, especially in industrial WSNs with dense node layouts or shared propagation paths, channels may exhibit spatial or temporal correlation due to factors like limited scattering or common obstacles. Such a correlation could alter the joint statistics of the channel gains $g_{k}={\left|h_{k}\right|}^{2}$ and potentially affect the optimal PS ratio distribution across relays. The investigation of correlated channel models and their impact on resource allocation is identified as an important direction for future work.

**Figure 2:**
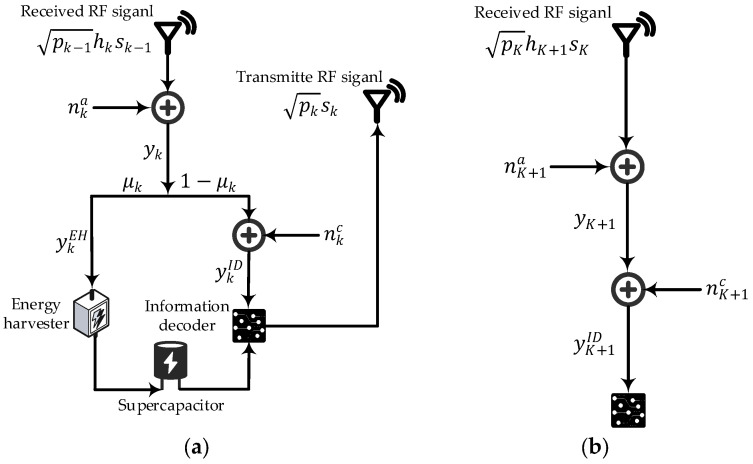
Signal processing flow at the relay and destination nodes: (**a**) For the relay nodes $r_{k}$, k∈{1,…,K}; (**b**) For the destination node $r_{K+1}$.

Figure 2 illustrates the signal processing flow at the relay and destination nodes in our multi-hop DF relay network model. As shown, the relay node $r_{k}$, k∈{1,…,K}, harvests energy from the $\mu_{k}$ portion of the received RF signal via an EH circuit and stores it in a supercapacitor. All harvested energy is utilized for ID and retransmission. This paper assumes that the source node $r_{0}$ possesses the channel state information (CSI) of all nodes, while each relay node $r_{k}$ and the destination node $r_{K+1}$ only have CSI of their corresponding communication channels, i.e., $h_{k}$, k∈{1,…,K} and $h_{K+1}$. It is assumed that direct links exist only between adjacent nodes. For example, there is no direct communication link between $r_{0}$ and $r_{2}$. Numerous sensor network routing algorithms support this assumption [20,31,32].

The RF signal received at node $r_{k}$, k∈{1,…,K+1}, from the previous-hop neighboring node $r_{k-1}$ is

(1) $$y_{k}=\sqrt{p_{k-1}}h_{k}s_{k-1}+n_{k}^{a},$$

where $s_{k-1}$ denotes the information symbol from the previous-hop neighboring node $r_{k-1}$, with each symbol having unit average power, i.e., $E\left[{\left|s_{k-1}\right|}^{2}\right]=1$. $p_{k-1}$ denotes the transmit power of $r_{k-1}$. As shown in Figure 2a, $n_{k}^{a}$ represents the additive white Gaussian noise (AWGN) introduced at the antenna of node $r_{k}$, which follows the distribution $n_{k}^{a}\sim CN\left(0,\delta^{2}\right)$. $\delta^{2}$ is the total power of $n_{k}^{a}$.

As shown in Figure 1 and Figure 2a, the received RF signal at relay node $r_{k}$, k∈{1,…,K}, is then split into two streams according to the PS ratio $\mu_{k}$, for EH and ID, respectively. The signal for EH can be expressed as

(2) $$y_{k}^{EH}=\sqrt{\mu_{k}}\left(\sqrt{p_{k-1}}h_{k}s_{k-1}+n_{k}^{a}\right).$$

The signal for ID at relay node $r_{k}$ can be expressed as

(3) $$y_{k}^{ID}=\sqrt{1-\mu_{k}}\left(\sqrt{p_{k-1}}h_{k}s_{k-1}+n_{k}^{a}\right)+n_{k}^{c},$$

where $n_{k}^{c}$ is the AWGN introduced by the ID circuit of node $r_{k}$, which follows the distribution $n_{k}^{c}\sim CN\left(0,\sigma^{2}\right)$. $\sigma^{2}$ is the total power of $n_{k}^{c}$.

We assume that all the harvested energy is used for ID and data retransmission. Thus, according to Equation (2), the transmit power of node $r_{k}$, k∈{1,…,K}, can be derived as

(4) $$p_{k}=\eta_{k}E\left[{\left|y_{k}^{EH}\right|}^{2}\right]=\eta_{k}\mu_{k}\left(p_{k-1}{\left|h_{k}\right|}^{2}+\delta^{2}\right),$$

where $\eta_{k}\in \left(0,1\right]$ represents the constant rectification efficiency factor at relay node $r_{k}$. This equation employs a linear EH model, where the harvested power is directly proportional to the received RF power, a common assumption in the initial theoretical analysis of SWIPT systems [8].

A more comprehensive discussion of EH models is warranted. While the linear model offers mathematical tractability, which is beneficial for deriving the fundamental closed-form solutions presented in this work, practical EH circuits exhibit non-linear characteristics. These non-linear models account for the saturation effect of the diodes in the rectifying circuit, where the harvested power plateaus when the input power exceeds a certain threshold, and the sensitivity effect, where EH only initiates once the input power surpasses a minimum activation level. Recent literature has extensively explored such practical non-linear EH models to capture more realistic system behaviors [33,34]. The linear model can be viewed as a special case of these non-linear models within a specific operating range where the input power is neither too low to cause sensitivity issues nor too high to trigger saturation. The primary focus of this paper is to establish the optimal resource allocation framework and derive the foundational closed-form solutions for the multi-hop DF relay network. The investigation of the proposed schemes under more complex and practical non-linear EH models, which introduce additional constraints and non-convexities, is a highly relevant and necessary direction for future work, as discussed in Section 5.

Since $p_{k-1}{\left|h_{k}\right|}^{2}\gg \delta^{2}$, we further obtain

(5) $$p_{k}\approx \eta_{k}\mu_{k}p_{k-1}g_{k}=p_{0}\prod_{i=1}^{k}\eta_{i}\mu_{i}g_{i},$$

where $g_{k}={\left|h_{k}\right|}^{2}$ represents the channel gain between nodes $r_{k-1}$ and $r_{k}$; $p_{0}$ denotes the transmit power of the source node $r_{0}$.

According to (3) and $E\left[{\left|s_{0}\right|}^{2}\right]=1$, the received signal-to-noise ratio (SNR) at relay $r_{1}$ can be expressed as

(6) $$\gamma_{1}=\frac{\left(1-\mu_{1}\right)p_{0}g_{1}}{\left(1-\mu_{1}\right)\delta^{2}+\sigma^{2}}.$$

In the PS-based SWIPT system, the additive noise at the antenna is generally much smaller than that introduced by the ID circuit [35,36]. Therefore, with $\delta^{2}\ll \sigma^{2}$, we further obtain

(7) $$\gamma_{1}=p_{0}A_{1}\frac{\left(1-\mu_{1}\right)}{\left(1-\mu_{1}\right)\delta^{2}/\sigma^{2}+1}\approx p_{0}A_{1}\left(1-\mu_{1}\right),$$

where $A_{1}=\frac{g_{1}}{\sigma^{2}}$.

Similarly, based on (3), (5) and $E\left[{\left|s_{k-1}\right|}^{2}\right]=1$, the received SNR at relay $r_{k}$, k∈{2,…,K}, is expressed as

(8) $$\gamma_{k}=\frac{\left(1-\mu_{k}\right)p_{k-1}g_{k}}{\left(1-\mu_{k}\right)\delta^{2}+\sigma^{2}}=p_{0}A_{k}\frac{\left(1-\mu_{k}\right)\prod_{i=1}^{k-1}\mu_{i}}{\left(1-\mu_{k}\right)\delta^{2}/\sigma^{2}+1}\approx {p_{0}A}_{k}\left(1-\mu_{k}\right)\prod_{i=1}^{k-1}\mu_{i},$$

where $A_{k}=\frac{\prod_{i=1}^{k}g_{i}\prod_{i=1}^{k-1}\eta_{i}}{\sigma^{2}}$.

As shown in Figure 1 and Figure 2b, since the destination node $r_{K+1}$ performs ID only without EH, the signal for ID at node $r_{K+1}$ can be expressed as

(9) $$y_{K+1}^{ID}=\sqrt{p_{K}}h_{K+1}s_{K}+n_{K+1}^{a}+n_{K+1}^{c}.$$

The received SNR at node $r_{K+1}$ is given by

(10) $$\gamma_{K+1}=\frac{p_{K}g_{K+1}}{\delta^{2}+\sigma^{2}}=p_{0}A_{K+1}\frac{\prod_{i=1}^{K}\mu_{i}}{\delta^{2}/\sigma^{2}+1}\approx p_{0}A_{K+1}\prod_{i=1}^{K}\mu_{i},$$

where $A_{K+1}=\frac{\prod_{i=1}^{K+1}g_{i}\prod_{i=1}^{K}\eta_{i}}{\sigma^{2}}$.

Since we obtained the SNR $\gamma_{k}$ for k∈{1,…,K+1}, the achievable rate for the k-th hop can be expressed as

(11) $$R_{k}=W\ln\left(1+\gamma_{k}\right).$$

where W denotes the transmission bandwidth.

Having experienced K hops, which means that the information transmission from the source node to the destination node requires K+1 frames, the end-to-end throughput of our multi-hop DF relay network can be expressed as

(12) $$R=\frac{min\left\{R_{1},\ldots .R_{K+1}\right\}}{K+1}.$$

In WSNs, given hardware costs, many sensor nodes transmit signals at a predetermined, constant power when the communication environment is stable and straightforward [37,38]. Thus, in this paper, the first problem we aim to address is to maximize the system throughput by optimizing the PS ratio $\mu_{k}$ of each relay under a given source node transmit power $p_{0}$. This optimization problem can be formulated as

(13a) $$\underset{\left\{\mu_{k}\right\}}{\max}R$$

(13b) $$s.t.\;0<\mu_{k}<1,\;k\in \left\{1,\ldots ,K\right\}.$$

Moreover, in WSNs, minimizing transmit power while ensuring QoS is essential for extending network lifetime and achieving green communication [39]. In this paper, we use the achievable rate as the metric for QoS. Therefore, the second problem we aim to solve is to minimize the source node transmit power $p_{0}$ by jointly optimizing the PS ratio $\mu_{k}$ of each relay and the source node transmit power $p_{0}$, under the constraint that the system throughput is no less than the required minimum achievable rate threshold. The corresponding second optimization problem is formulated as follows:

(14a) $$\underset{\left\{\mu_{k}\right\},\;p_{0}}{\min}p_{0}$$

(14b) $$s.t.\;0<\mu_{k}<1,\;k\in \left\{1,\ldots ,K\right\},$$

(14c) $$p_{min}\leq p_{0}\leq p_{max},$$

(14d) R≥Q,

where $p_{min}$ and $p_{max}$ represent the minimum and maximum transmit power of the source node $r_{0}$, respectively, and Q denotes the minimum achievable rate threshold of the system.

## 3. Multi-Hop Relaying Joint Optimization

This section addresses the STM problem under a given source node transmit power, i.e., (13), in Section 3.1, and the SPM problem under a given minimum QoS requirement, i.e., (14), in Section 3.2, respectively. Additionally, we discuss the real-world implementation of our proposed solutions to the aforementioned two problems.

### 3.1. System Throughput Maximization

First, Problem (13) is non-convex with respect to the variables $\left\{\mu_{k}\right\}$. This can be demonstrated by computing and analyzing the Hessian matrix of $R_{k}$. However, due to the complexity of the computational process, we omit it in this paper. In standard convex optimization problems, since any local minimum is also the global minimum, and the optimal solution can be easily found using methods such as Lagrangian duality [40,41], in this subsection, we first attempt to transform Problem (13) into a standard convex optimization problem. To overcome the non-differentiability of $min\left\{R_{1},\ldots .R_{K+1}\right\}$ in (13a), we introduce an auxiliary variable $r=\frac{min\left\{R_{1},\ldots .R_{K+1}\right\}}{W}$, transforming the complex min–max problem into a standard constrained optimization problem as follows.

(15a) $$\underset{\left\{\mu_{k}\right\},r}{\max}r$$

(15b) $$s.t.\;\ln\left(1+B_{1}\left(1-\mu_{1}\right)\right)\geq r,$$

(15c) $$\ln\left(1+B_{k}\left(1-\mu_{k}\right)\prod_{i=1}^{k-1}\mu_{i}\right)\geq r,\;k\in \left\{2,\ldots ,K\right\},$$

(15d) $$\ln\left(1+B_{K+1}\prod_{i=1}^{K}\mu_{i}\right)\geq r,$$

(15e) $$0<\mu_{k}<1,\;k\in \left\{1,\ldots ,K\right\}.$$

where $B_{k}={p_{0}A}_{k}$, k∈{1,…,K+1}.

Let $x_{k}=1-\mu_{k}$ and $y=\ln\left(e^{r}-1\right)$. After taking the exponential, rearranging terms, and taking the logarithm of (15b), (15c), and (15d), we obtain

(16a) $$\underset{\left\{x_{k}\right\},y}{\min}-y$$

(16b) $$s.t.\;y-\ln x_{1}-\ln B_{1}\leq 0,$$

(16c) $$y-\sum_{i=1}^{k-1}\ln\left(1-x_{i}\right)-\ln x_{k}-\ln B_{k}\leq 0,\;k\in \left\{2,\ldots ,K\right\},$$

(16d) $$y-\sum_{i=1}^{K}\ln\left(1-x_{i}\right)-\ln B_{K+1}\leq 0,$$

(16e) $$0<x_{k}<1,k\in \left\{1,\ldots ,K\right\}.$$

The transformed problem (16) is a convex optimization problem, as proved below.

**Proposition** **1.** *Problem (16) is a convex optimization problem.*

**Proof of Proposition** **1.** Since −y is a linear function and linear functions are both convex and concave, the objective function is convex. (16e) is a convex set. Therefore, to prove that problem (16) is a convex problem, it suffices to show that the corresponding functions below for constraints (16b–d) are convex.

(17) $$f_{1}\left(x_{1},y\right)=y-\ln x_{1}-\ln B_{1},$$

(18) $$f_{k}\left(x_{1},\ldots ,x_{k},y\right)=y-\sum_{i=1}^{k-1}\ln\left(1-x_{i}\right)-\ln x_{k}-\ln B_{k},\;k\in \left\{2,\ldots ,K\right\},$$

(19) $$f_{K+1}\left(x_{1},\ldots ,x_{K},y\right)=y-\sum_{i=1}^{K}\ln\left(1-x_{i}\right)-\ln B_{K+1}.$$

Define the auxiliary function u(x)=−lnx. Its second derivative is $u^{\prime \prime }\left(x\right)=1/x^{2}>0$. Therefore, u(x) is a convex function. Moreover, since y is a linear term and $\ln B_{1}$ is a constant, then $f_{1}\left(x_{1},y\right)$ is the sum of convex functions and is also a convex function.

Define the auxiliary function v(x)=−ln(1−x). Its second derivative is $v^{\prime \prime }\left(x\right)=1/{\left(1-x\right)}^{2}>0$. Therefore, v(x) is a convex function. Similarly, both $f_{k}\left(x_{1},\ldots ,x_{k},y\right)$ and $f_{K+1}\left(x_{1},\ldots ,x_{K},y\right)$ are sums of convex functions and are thus also convex functions.

Therefore, (16) is a convex problem. □

Thus, the optimal solution to problem (16) satisfies the Karush–Kuhn–Tucker (KKT) conditions, and if and only if Slater’s condition holds, the KKT point is the global optimum. That is, at the optimal solution, constraints (16b–d) must be tight (i.e., equality holds) [40,41]. By setting (16b–d) equal and making slight rearrangements, we obtain

(20) $$B_{1}x_{1}=e^{y},$$

(21) $$B_{k}x_{k}\prod_{i=1}^{k-1}\left(1-x_{i}\right)=e^{y},\;k\in \left\{2,\ldots ,K\right\},$$

(22) $$B_{K+1}\prod_{i=1}^{K}\left(1-x_{i}\right)=e^{y}.$$

The solution for $x_{k}$, k∈{1,…,K}, can be given in the following three cases.

Case 1: The expression for $x_{K}$.

By solving the case for k=K in Equations (21) and (22) simultaneously, we obtain

(23) $$B_{K}x_{K}\prod_{i=1}^{K-1}\left(1-x_{i}\right)=B_{K+1}\prod_{i=1}^{K}\left(1-x_{i}\right).$$

After rearrangement, we obtain

(24) $$x_{K}=\frac{A_{K+1}}{A_{K}+A_{K+1}}.$$

Case 2: The expression for $x_{k}$, k∈{2,…,K−1}.

By simultaneously solving the cases for k and k+1 in Equation (21), we obtain

(25) $$B_{k}x_{k}\prod_{i=1}^{k-1}\left(1-x_{i}\right)=B_{k+1}x_{k+1}\prod_{i=1}^{k}\left(1-x_{i}\right).$$

After rearrangement, we obtain

(26) $$x_{k}=\frac{A_{k+1}x_{k+1}}{A_{k}+A_{k+1}x_{k+1}}.$$

Case 3: The expression for $x_{1}$.

By simultaneously solving Equation (20) and the case of k=2 in Equation (21), we can obtain

(27) $$B_{1}x_{1}=B_{2}x_{2}\left(1-x_{1}\right).$$

After rearrangement, we obtain

(28) $$x_{1}=\frac{A_{2}x_{2}}{A_{1}+A_{2}x_{2}}.$$

To find the optimal PS ratios $\left\{\mu_{k}^{*}\right\}$, we introduce the reciprocal variables $z_{k}=1/x_{k}$, yielding the following:

For k=K, we have

(29) $$z_{K}=1+\frac{A_{K}}{A_{K+1}}.$$

For k∈{1,…,K−1}, we have

(30) $$z_{k}=1+\frac{A_{k}}{A_{k+1}}z_{k+1}.$$

Define $z_{K+1}=1$, then the recurrence relation of Equation (30) also holds for k=K, and Equations (29) and (30) can be unified as

(31) $$z_{k}=\{\begin{array}{c} 1+\frac{A_{k}}{A_{k+1}}z_{k+1},\;\;k\in \left\{1,\ldots ,K\right\}\\ 1,\;\;k=K+1 \end{array}.$$

Next, we prove the following proposition:

**Proposition** **2.** *The closed-form expression for *$z_{k}$ *is*

(32) $$z_{k}=A_{k}\sum_{i=k}^{K+1}\frac{1}{A_{i}},k\in \left\{1,\ldots ,K+1\right\}.$$

**Proof of Proposition** **2.** First, it is straightforward to verify the case when k=K+1 as follows:

(33) $$z_{K+1}=A_{K+1}\sum_{i=K+1}^{K+1}\frac{1}{A_{i}}=1.$$

Assume that

(34) $$z_{k+1}=A_{k+1}\sum_{i=k+1}^{K+1}\frac{1}{A_{i}},\;k\in \left\{1,\ldots ,K\right\}$$

holds. According to the recurrence relation (31), we have

(35) $$z_{k}=1+\frac{A_{k}}{A_{k+1}}\left(A_{k+1}\sum_{i=k+1}^{K+1}\frac{1}{A_{i}}\right)=A_{k}\left(\frac{1}{A_{k}}+\sum_{i=k+1}^{K+1}\frac{1}{A_{i}}\right)=A_{k}\sum_{i=k}^{K+1}\frac{1}{A_{i}}.$$

Therefore, by mathematical induction, we can prove that (32) holds. □

According to (35), $x_{k}=1/z_{k}$ and $\mu_{k}=1-x_{k}$, we can obtain the closed-form solution for the optimal PS combination that maximizes the system throughput under a given source node transmit power as follows:

(36) $$\mu_{k}^{*}=1-\frac{1}{A_{k}\sum_{i=k}^{K+1}\frac{1}{A_{i}}},\;k\in \left\{1,\ldots ,K\right\}.$$

### 3.2. Source Power Minimization

Similar to Problem (13), due to the presence of R in constraint (14d), Problem (14) is also a non-convex optimization problem with respect to the decision variables $\left\{\mu_{k}\right\}$ and $p_{0}$. We first simplify constraint (14d) and arrive at

(37) $$R_{k}\geq \left(K+1\right)Q,\;k\in \left\{1,\ldots ,K+1\right\}.$$

Combining with Equation (11), we can further obtain

(38) $$\gamma_{k}\geq e^{\left(K+1\right)Q/W}-1,k\in \left\{1,\ldots ,K+1\right\}.$$

By combining Equations (7), (8) and (10), Constraint (14d) can be written separately as

(39) $$p_{0}\left(1-\mu_{1}\right)\geq C_{1},$$

(40) $$p_{0}\left(1-\mu_{k}\right)\prod_{i=1}^{k-1}\mu_{i}\geq C_{k},\;k\in \left\{2,\ldots ,K\right\},$$

(41) $$p_{0}\prod_{i=1}^{K}\mu_{i}\geq C_{K+1},$$

where $C_{k}=\frac{e^{\left(K+1\right)Q/W}-1}{A_{k}}$, k∈{1,…,K+1}.

Taking the logarithm on both sides of inequalities (39)–(41), Problem (14) can be equivalently transformed into

(42a) $$\underset{\left\{\mu_{k}\right\},\;p_{0}}{\min}p_{0}$$

(42b) $$s.t.\;0<\mu_{k}<1,\;k\in \left\{1,\ldots ,K\right\},$$

(42c) $$p_{min}\leq p_{0}\leq p_{max},$$

(42d) $$\ln C_{1}-\ln p_{0}-\ln\left(1-\mu_{1}\right)\leq 0,$$

(42e) $$\ln C_{k}-\ln p_{0}-\ln\left(1-\mu_{k}\right)-\sum_{i=1}^{k-1}\ln\mu_{i}\leq 0,\;k\in \left\{2,\ldots ,K\right\},$$

(42f) $$\ln C_{K+1}-\ln p_{0}-\sum_{i=1}^{K}\ln\mu_{i}\leq 0.$$

This transformed problem can be proven to be a convex optimization problem as follows:

**Proposition** **3.** *Problem (42) is a convex optimization problem.*

**Proof of Proposition** **3.** First, the objective function $p_{0}$ is a linear function and is therefore convex. Second, the constraints (42b) and (42c) are linear inequalities, which define convex sets. Consequently, we only need to prove that the following functions corresponding to constraints (42d–f) are convex.

(43) $$g_{1}\left(\mu_{1},p_{0}\right)=\ln C_{1}-\ln p_{0}-\ln\left(1-\mu_{1}\right),$$

(44) $$g_{k}\left(\mu_{1},\ldots ,\mu_{k},p_{0}\right)=\ln C_{k}-\ln p_{0}-\ln\left(1-\mu_{k}\right)-\sum_{i=1}^{k-1}\ln\mu_{i},\;k\in \left\{2,\ldots ,K\right\},$$

(45) $$g_{K+1}\left(\mu_{1},\ldots ,\mu_{K},p_{0}\right)=\ln C_{K+1}-\ln p_{0}-\sum_{i=1}^{K}\ln\mu_{i}.$$

Since we have already demonstrated in the proof of Proposition 1 that the auxiliary functions u(x)=−lnx and v(x)=−ln(1−x) are convex, it follows that $-\ln p_{0}$, $-\ln\left(1-\mu_{1}\right)$, $-\ln\left(1-\mu_{k}\right)$, $-\sum_{i=1}^{k-1}\ln\mu_{i}$, and $-\sum_{i=1}^{K}\ln\mu_{i}$ are all convex functions. The functions $g_{k}$, where k∈{1,…,K+1}, are sums of constants and these convex functions, and thus, are also convex.

In summary, all constraints define convex sets, and the objective function is convex. Therefore, (42) is a convex optimization problem. □

Since the problem (42) is a convex optimization problem, it can be solved via the KKT conditions. Boundary constraints (42b) and (42c) are treated as the domain of the variables and are not active. If they are not active, then constraints (42d–f) are tight (i.e., they hold with equality) at the optimal solution [40,41]. By setting (42d–f) equal and making slight rearrangements, we obtain

(46) $$p_{0}\left(1-\mu_{1}\right)=C_{1},$$

(47) $$p_{0}\left(1-\mu_{k}\right)\prod_{i=1}^{k-1}\mu_{i}=C_{k},\;k\in \left\{2,\ldots ,K\right\},$$

(48) $$p_{0}\prod_{i=1}^{K}\mu_{i}=C_{K+1},$$

The solution for $\mu_{k}$, k∈{1,…,K}, can be given in the following three cases.

Case 1: The expression for $\mu_{K}$.

By solving the case for k=K in Equations (47) and (48) simultaneously, we can obtain

(49) $$\frac{p_{0}\left(1-\mu_{K}\right)\prod_{i=1}^{K-1}\mu_{i}}{C_{K}}=\frac{p_{0}\prod_{i=1}^{K}\mu_{i}}{C_{K+1}}.$$

After rearrangement, we obtain

(50) $$\mu_{K}=\frac{C_{K+1}}{C_{K}+C_{K+1}}=\frac{A_{K}}{A_{K}+A_{K+1}}.$$

Case 2: The expression for $\mu_{k}$, k∈{2,…,K−1}.

By simultaneously solving the cases for k and k+1 in Equation (47), we obtain

(51) $$\frac{p_{0}\left(1-\mu_{k}\right)\prod_{i=1}^{k-1}\mu_{i}}{C_{k}}=\frac{p_{0}\left(1-\mu_{k+1}\right)\prod_{i=1}^{k}\mu_{i}}{C_{k+1}}.$$

After rearrangement, we obtain

(52) $$\mu_{k}=\frac{C_{k+1}}{C_{k}\left(1-\mu_{k+1}\right)+C_{k+1}}=\frac{A_{k}}{A_{k+1}\left(1-\mu_{k+1}\right)+A_{k}}.$$

Case 3: The expression for $\mu_{1}$.

By simultaneously solving Equation (46) and the case of k=2 in Equation (47), we can obtain

(53) $$\frac{p_{0}\left(1-\mu_{1}\right)}{C_{1}}=\frac{p_{0}\left(1-\mu_{2}\right)\mu_{1}}{C_{2}}.$$

After rearrangement, we obtain

(54) $$\mu_{1}=\frac{C_{2}}{C_{1}\left(1-\mu_{2}\right)+C_{2}}=\frac{A_{1}}{A_{2}\left(1-\mu_{2}\right)+A_{1}}.$$

Equations (50), (52) and (54) can be unified as

(55) $$\mu_{k}=\{\begin{array}{c} \frac{A_{k}}{A_{k+1}\left(1-\mu_{k+1}\right)+A_{k}},\;\;k\in \left\{1,\ldots ,K-1\right\}\\ \frac{A_{K}}{A_{K}+A_{K+1}},\;\;k=K \end{array}.$$

Similar to Proposition 2, we can prove the following proposition.

**Proposition** **4.** *The closed-form expression for *$\mu_{k}$ *is*

(56) $$\mu_{k}=1-\frac{1}{A_{k}\sum_{i=k}^{K+1}\frac{1}{A_{i}}},k\in \left\{1,\ldots ,K\right\}.$$

**Proof of Proposition** **4.** We first verify the case when k=K as follows:

(57) $$\mu_{K}=1-\frac{1}{A_{K}\left(\frac{1}{A_{K}}+\frac{1}{A_{K+1}}\right)}=\frac{A_{K}}{A_{K}+A_{K+1}}.$$

Clearly, (57) is consistent with (55). Assume that

(58) $$\mu_{k+1}=1-\frac{1}{A_{k+1}\sum_{i=k+1}^{K+1}\frac{1}{A_{i}}},\;k\in \left\{1,\ldots ,K-1\right\}$$

holds. Substituting (58) into the recurrence relation (55), we obtain

(59) $$\mu_{k}=\frac{A_{k}}{\frac{1}{\sum_{i=k+1}^{K+1}\frac{1}{A_{i}}}+A_{k}}=\frac{A_{k}}{\frac{\frac{A_{k}}{A_{k}}+A_{k}\sum_{i=k+1}^{K+1}\frac{1}{A_{i}}}{\sum_{i=k+1}^{K+1}\frac{1}{A_{i}}}}=\frac{A_{k}}{\frac{A_{k}\sum_{i=k}^{K+1}\frac{1}{A_{i}}}{\sum_{i=k+1}^{K+1}\frac{1}{A_{i}}}}=\frac{\sum_{i=k}^{K+1}\frac{1}{A_{i}}-\frac{1}{A_{k}}}{\sum_{i=k}^{K+1}\frac{1}{A_{i}}}=1-\frac{1}{A_{k}\sum_{i=k}^{K+1}\frac{1}{A_{i}}}.$$

Therefore, we prove that (56) holds. □

Now, we proceed to derive the closed-form expression for $p_{0}$. First, we add Equations (46)–(48) to obtain

(60) $$p_{0}\left\{\left(1-\mu_{1}\right)+\sum_{k=2}^{K}\left[\left(1-\mu_{k}\right)\prod_{i=1}^{k-1}\mu_{i}\right]+\prod_{i=1}^{K}\mu_{i}\right\}=\sum_{i=1}^{K+1}C_{k}.$$

Let $S=\left(1-\mu_{1}\right)+\sum_{k=2}^{K}\left[\left(1-\mu_{k}\right)\prod_{i=1}^{k-1}\mu_{i}\right]+\prod_{i=1}^{K}\mu_{i}$. Expanding the last two terms of S, we have

(61) $$S=\left(1-\mu_{1}\right)+\left(1-\mu_{2}\right)\mu_{1}+\left(1-\mu_{3}\right)\mu_{1}\mu_{2}+\cdots +\left(1-\mu_{K}\right)\mu_{1}\mu_{2}\cdots \mu_{K-1}+\mu_{1}\mu_{2}\cdots \mu_{K}.$$

This is a telescoping sum, where all the intermediate terms cancel each other out, resulting in S=1. Therefore, we obtain

(62) $$p_{0}=\sum_{i=1}^{K+1}C_{k}.$$

Equations (56) and (62) provide the closed-form optimal solutions for $\mu_{k}$ and $p_{0}$, respectively, derived without accounting for the linear constraints (42b) and (42c). According to (56), it is evident that the solution for $\mu_{k}$ satisfies constraint (42b).

Now, consider the scenario where the boundary constraint (42c) is activated, and discuss it in three cases:

Case 1: $\sum_{i=1}^{K+1}C_{k}<p_{min}$.

In this case, the unbounded solution violates the lower-bound constraint $p_{0}\geq p_{min}$. Therefore, the optimal solution should be set to $p_{min}$. At this point, $\mu_{k}$ is chosen according to Equation (36) to yield the solution that maximizes the system throughput. Note that (36) is consistent with (56). Thus, the optimal solution is

(63) $$p_{0}^{*}=p_{min},$$

(64) $$\mu_{k}^{*}=1-\frac{1}{A_{k}\sum_{i=k}^{K+1}\frac{1}{A_{i}}},\;k\in \left\{1,\ldots ,K\right\}.$$

Case 2: $\sum_{i=1}^{K+1}C_{k}>p_{max}$.

In this case, the unbounded solution violates the upper-bound constraint $p_{0}\leq p_{max}$, rendering the problem infeasible with no solution.

Case 3: $p_{min}\leq \sum_{i=1}^{K+1}C_{k}\leq p_{max}$.

In this case, the unbounded solution satisfies the boundary constraints, so the optimal solution is the unbounded one:

(65) $$p_{0}^{*}=\sum_{i=1}^{K+1}C_{k},$$

(66) $$\mu_{k}^{*}=1-\frac{1}{A_{k}\sum_{i=k}^{K+1}\frac{1}{A_{i}}},\;k\in \left\{1,\ldots ,K\right\}.$$

### 3.3. Implementation Analysis of the Proposed Schemes

In this section, we analyze the specific implementation plans for the proposed STM scheme, which is based on the closed-form optimal solution from Section 3.1, and the proposed SPM scheme, which is based on the closed-form optimal solution from Section 3.2.

First, for the proposed STM scheme, based on Equation (36), it is evident that $\mu_{k}^{*}$ can be obtained in a centralized manner by the source node, which first acquires the CSI $h_{k}$, k∈{1,…,K+1} for each hop across the entire relay network and then calculates the auxiliary variable $A_{k}$.

On the other hand, according to Equation (31) and the expressions for the auxiliary variables $A_{k}$, k∈{1,…,K+1}, we combine (31) with $\mu_{k}=1-1/z_{k}$ to obtain

(67) $$\mu_{k}=\{\begin{array}{c} \frac{1}{1+g_{k+1}\eta_{k}\left(1-\mu_{k+1}\right)},\;\;k\in \left\{1,\ldots ,K-1\right\}\\ \frac{1}{g_{K+1}\eta_{K}},\;\;k=K \end{array}.$$

Therefore, the relay node can obtain its own $\mu_{k}^{*}$ in a distributed manner by utilizing the local CSI $h_{k+1}$ and the optimal PS ratio $\mu_{k+1}^{*}$ of the next-hop adjacent relay.

For the proposed SPM scheme, Equation (55) similarly allows us to derive Equation (67). Therefore, $\mu_{k}^{*}$ can also be obtained either in a centralized or distributed manner.

However, according to Equation (65) and the expressions for $C_{k}$, k∈{1,…,K+1}, it is evident that the calculation of $p_{0}^{*}$ requires the source node to possess global CSI and be computed in a centralized manner.

## 4. Simulation Results and Discussion

In this section, we conduct numerical simulations to evaluate the performance of the two proposed schemes based on the closed-form optimal solutions presented in Section 3, within the SWIPT-enabled multi-hop DF relaying system. The numerical simulations are performed on a laptop with an AMD Ryzen R9 7945HX CPU and 64 GB of RAM. The algorithms are implemented, and the data is analyzed using MATLAB R2022b as the software platform.

Unless otherwise specified, the simulations in this section adopt the following parameter settings: the power spectral density of the AWGN introduced at the ID circuit of node $r_{k}$, k∈{1,…,K+1}, is $\frac{\sigma^{2}}{W}=-114$ dBm/Hz; the transmission bandwidth is W=1 MHz; the rectification efficiency at each relay node is uniformly set to $\eta_{k}=0.95$, k∈{1,…,K}; the minimum transmit power of the source node $r_{0}$ is $p_{min}=20$ dBm; the maximum transmit power of the source node $r_{0}$ is $p_{max}=40$ dBm.

**Figure 3:**
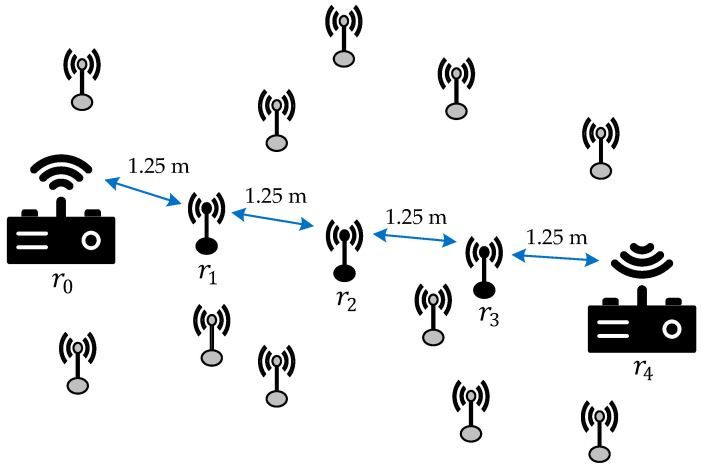
An example of the network topology used in the simulations when K=3.

Additionally, we assume that in the considered multi-hop relay network system, the distance between two nodes in each hop is equal, and the sum of these distances is 5 m. Specifically, if there are K relays in our model, the multi-hop relay network consists of K+1 hops, with the spacing of each hop being $\frac{5}{K+1}$ m. Figure 3 provides an example for the case of K=3.

The channel model used in the simulations considers both large-scale fading and small-scale fading. For large-scale fading, the log-distance path loss model is adopted, with a path loss exponent set to 3.8, a carrier frequency of 2.4 GHz, and a reference distance of 1 m. Regarding the small-scale fading model, we adopt Rician fading to accurately represent the propagation environment. Given that the relays are typically deployed in proximity within controlled settings (e.g., industrial halls), a strong LoS path is expected between communicating nodes. Therefore, the channel gain $g_{k}$, k∈{1,…,K+1}, is modeled as follows: a Rician distribution with a K-factor of 7, representing a dominant LoS component relative to the scattered multipath components. The channel gains are modeled as independent Rician variables to isolate the effects of multi-hop resource allocation. While this assumes ideal uncorrelated conditions, it provides a baseline for evaluating the proposed schemes; correlated channels would require additional analysis beyond the scope of this paper.

All simulation results are obtained by averaging over 1000 random channel realizations.

### 4.1. System Throughput Maximization

We compare the proposed STM scheme based on the closed-form solution from Section 3.1 with the following benchmark schemes: the first is the performance-optimal scheme based on exhaustive search, which determines the optimal PS ratio combination using an exhaustive search with a PS ratio step size of 0.02; the other three suboptimal schemes fix the PS ratio at each relay to 0.25, 0.5, and 0.75, respectively.

#### 4.1.1. System Throughput vs. Source Transmit Power

**Figure 4:**
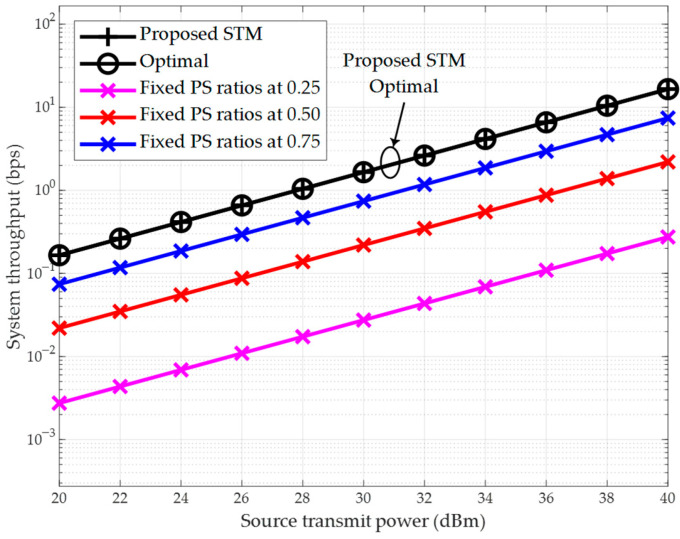
System throughput vs. source transmit power (K=3).

First, Figure 4 illustrates the relationship between throughput and the source transmit power in our SWIPT-enabled multi-hop DF relaying system, where the number of hops is set to K=3. Accordingly, the distance per hop is calculated as $\frac{5}{K+1}=1.25$ m. As shown in this figure, the throughput of all schemes increases with the rise in the source transmit power. For example, the throughput of the proposed scheme increases from $1.65\times 10^{-1}$ bps at $p_{0}=p_{min}$ to $1.65\times 10^{2}$ bps at $p_{0}=p_{max}$. Furthermore, the curve of the proposed scheme based on the closed-form solution from Section 3.1 coincides with that of the exhaustive search-based scheme. This consistency is also observed under other parameter settings, demonstrating that the proposed scheme achieves optimal throughput performance. On the other hand, the proposed scheme significantly outperforms the three suboptimal schemes with fixed PS ratios in terms of throughput. For instance, the results indicate that the average throughput of the proposed scheme is 123% higher than that of the scheme with fixed PS ratios of 0.75. This notable advantage persists even when the fixed PS ratios are set to other values.

#### 4.1.2. System Throughput vs. Number of Relays

**Figure 5:**
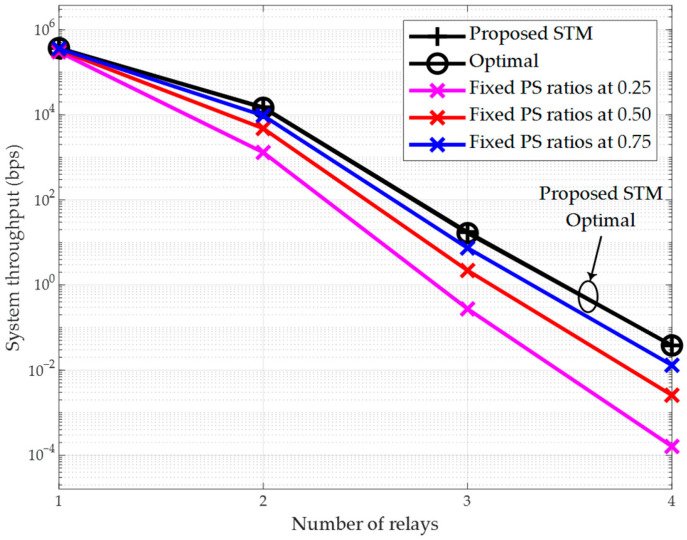
System throughput vs. number of relays ($p_{0}=p_{max}$).

Figure 5 illustrates the relationship between the throughput of the multi-hop relay system and the number of relays, K. The transmit power of the source node is set to $p_{0}=p_{max}$. The number of relays in the multi-hop relay system increases from 1 to 4, corresponding to an increase in the number of hops between the source and destination nodes from 2 to 5. Given that the distance per hop is equal and the total distance of all hops is 5 m, the per-hop distances are 2.5 m, 1.67 m, 1.25 m, and 1 m, respectively. The conclusions from Figure 5 align with those from Figure 4: the proposed scheme, based on the closed-form solution from Section 3.1, achieves optimal performance, and its throughput surpasses that of the suboptimal schemes with fixed PS ratios. For instance, when the number of relays is K=3, the throughput of the proposed scheme matches that of the optimal scheme at $1.65\times 10^{2}$ bps, whereas the throughput of the scheme with fixed PS ratios of 0.75 is only 7.41 bps.

Furthermore, it can be observed that the throughput of all schemes decreases rapidly as the number of relays increases. Taking the proposed scheme as an example, when K=1, the corresponding throughput is $3.70\times 10^{5}$ bps; however, when K=4, its throughput drops to a negligible $3.80\times 10^{-2}$ bps. The throughput decline in multi-hop scenarios reflects the cumulative effect of practical EH constraints, where harvested energy per relay diminishes with hop distance. This indicates that when deploying a multi-hop relay network, the number of relays must be controlled, provided that favorable channel conditions can be achieved for each hop. Nevertheless, we can still observe that the proposed scheme not only outperforms the suboptimal schemes with fixed PS ratios but also exhibits a significantly slower rate of throughput degradation as the number of relays K increases from 1 to 4, compared to the fixed PS ratio schemes. All the above phenomena demonstrate the performance advantages of the proposed scheme.

#### 4.1.3. Insights on Relay Number and Placement

The results presented in Figure 5 demonstrate that the system throughput decreases significantly as the number of relays (and thus hops) increases. This subsection provides a deeper analysis of this trend and derives practical insights for network design.

The primary reason for the throughput degradation with increasing hop count is the cumulative effect of PS and signal attenuation at each relay. In the considered PS-based SWIPT scheme, each relay node diverts a portion $\mu_{k}$ of the received power for EH, which reduces the power available for information transmission to the next hop. Although the closed-form solution optimizes $\mu_{k}$ to balance this trade-off, the multiplicative nature of power transfer over multiple hops (as seen in Equations (5) and (10)) inherently leads to a reduction in the signal power reaching the destination. Furthermore, each hop introduces additional channel attenuation and potential decoding errors in DF relays, which collectively constrain the end-to-end throughput governed by the min-hop rate in Equation (12).

This analysis reveals a critical trade-off in multi-hop SWIPT network design: increasing the number of relays extends the communication coverage by breaking a long, low-quality link into several short, high-quality links, but at the cost of reduced overall throughput due to the cumulative losses. Therefore, determining the optimal number of relays is essential.

Based on our simulations for a total distance of 5 m, the following insights can be drawn:

1. For applications requiring very high throughput over short distances (e.g., within a single room), a minimal number of relays (e.g., K=1 or 2) is preferable. For instance, with K=1, the throughput is $3.70\times 10^{5}$ bps, which is suitable for high-data-rate sensor networks.
2. For applications prioritizing coverage extension over raw speed (e.g., monitoring a long corridor), a larger number of relays (e.g., K=3 or 4) can be used, accepting a lower throughput. However, as shown, the throughput for K=4 drops to nearly zero, indicating a practical limit exists.
3. An optimal point often exists. In our scenario, K=2 (3 hops) provides a balance, maintaining a throughput of approximately ${1.46\times 10}^{4}$ bps while significantly extending the range compared to direct transmission.

Regarding relay placement, our model assumes equal hop distances for analytical tractability. However, the findings suggest that in a real-world deployment, relays should be placed such that the channel gains $g_{k}$ for each hop are maximized and balanced. Avoiding hops with severe path loss (e.g., due to obstacles) is critical. The proposed optimal PS ratio solution (Equation (36)) dynamically allocates resources based on channel conditions; hence, placing relays in locations with strong LoS components (high Rician K-factor) can further enhance performance. Ultimately, the number and placement of relays should be jointly optimized based on the specific environment and QoS requirements, a promising direction for future work involving spatial optimization algorithms.

#### 4.1.4. Computational Time vs. Number of Relays

**Figure 6:**
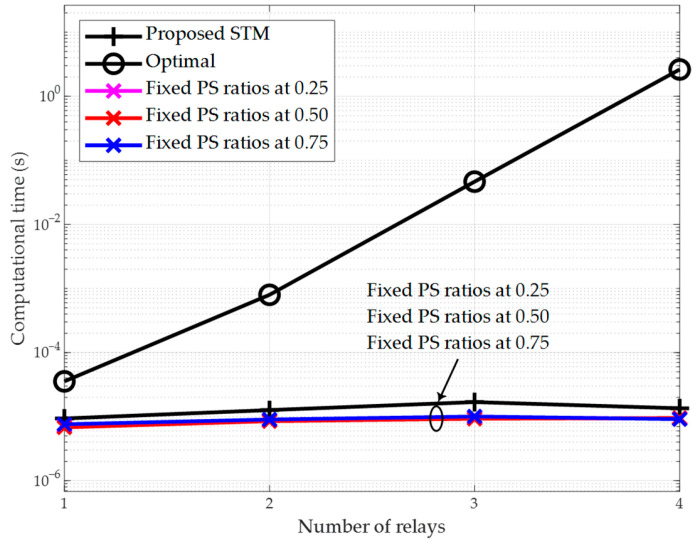
Computational time vs. number of relays ($p_{0}=p_{max}$).

Figure 4 and Figure 5 demonstrate that the proposed scheme achieves optimal throughput performance. Next, in Figure 6, we evaluate the performance of the proposed STM scheme from the perspective of computational efficiency. For this purpose, we measure the computational time of all schemes. The transmit power of the source node is set to $p_{0}=p_{max}$. The results show that the exhaustive search-based optimal scheme requires the longest computational time, which increases rapidly with the number of relays. When the number of relays K=4, the optimal scheme takes 2.62 s to complete the computation, which is unacceptable for practical communication systems.

It is worth noting that in our implementation of the optimal scheme based on the exhaustive search, we did not use nested loops. Instead, we utilized MATLAB’s built-in “ndgrid” function to generate a grid matrix of all possible PS ratio combinations, thereby accelerating the search process. This approach trades increased computational memory usage for a significant reduction in computation time. However, when the number of relays K is large, the computation time remains unsatisfactory and can lead to a memory explosion issue.

On the other hand, as expected, the three schemes with fixed PS ratios exhibit identical and minimal computational times, which remain almost constant as the number of relays increases. In comparison, the computational time of the proposed STM scheme is within the same order of magnitude as these three suboptimal schemes. For instance, when K=3, the computational time of the proposed STM scheme is $1.68\times 10^{-5}$ s, which is only 168% of that of the suboptimal scheme with fixed PS ratios of 0.75. Evidently, the proposed STM scheme is well-suited for practical communication systems.

The proposed SPM scheme shares the same computational complexity as the proposed STM scheme, as both are derived from the identical closed-form solution.

To further demonstrate the computational efficiency of the proposed schemes, we compare their complexity with the existing iterative method in [25], which addresses a similar multi-hop DF relay network but employs TS-SWIPT. The scheme in [25] uses an interior-point algorithm with a complexity of $O\left({\left(K+1\right)}^{3.5}\log\frac{1}{\epsilon_{o}}\right)$, where $\epsilon_{o}$ is the tolerance. This complexity scales polynomially with the number of relays K, making it less suitable for large-scale networks. In contrast, our proposed STM and SPM schemes leverage closed-form solutions derived from convex optimization, which require only direct evaluation of analytical expressions (e.g., Equations (36) and (56)). Thus, their computational complexity is O(1), i.e., constant time, regardless of K. This fundamental difference is evident in Figure 6, where the computational time of our schemes remains flat as K increases, while iterative methods like [25] would exhibit significant growth. The O(1) complexity ensures real-time applicability in resource-constrained IoT scenarios.

### 4.2. Source Power Minimization

In this section, we evaluate the performance of the SPM scheme based on the closed-form solution from Section 3.2. Similarly, our comparative algorithms include the optimal scheme derived from exhaustive search and suboptimal schemes that utilize fixed PS ratios along with a fixed source node transmit power. The difference lies in the configuration of the exhaustive search: besides setting the PS ratio step size to 0.02, we also set the source node transmit power search step size to 1 dB. Furthermore, for the three suboptimal schemes, the PS ratios remain fixed at 0.25, 0.5, and 0.75, respectively, and the source node transmit power is fixed at 30 dBm.

**Figure 7:**
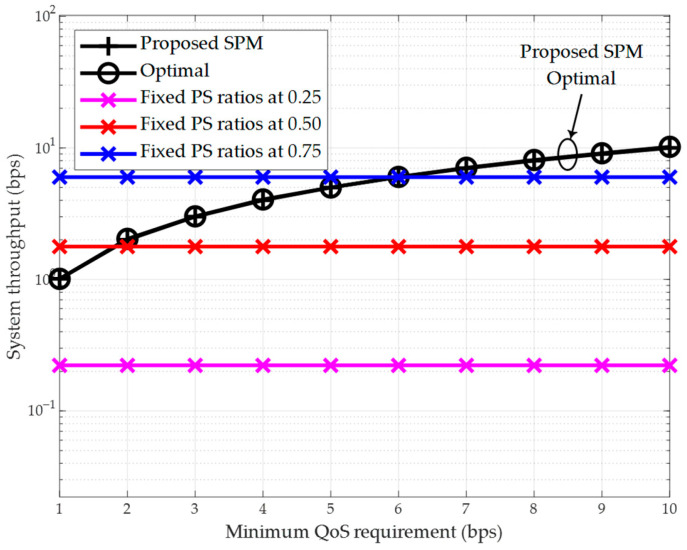
System throughput vs. minimum QoS requirement (K=3).

**Figure 8:**
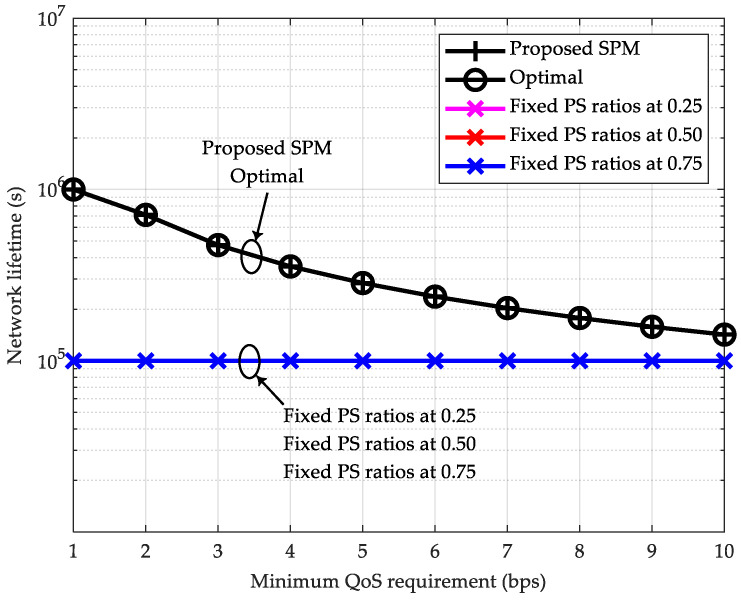
Network lifetime vs. minimum QoS requirement (K=3).

Since the proposed SPM scheme based on the closed-form solution from Section 3.2 demonstrates performance trends and characteristics similar to those of the proposed STM scheme from Section 3.1 in terms of system throughput versus the number of relays and computational time versus the number of relays, the corresponding simulation result figures are not presented separately. Instead, Figure 7 and Figure 8 are utilized to highlight the performance advantages of the proposed SPM scheme in guaranteeing the system’s minimum QoS requirement and extending the network lifetime.

#### 4.2.1. System Throughput vs. Minimum QoS Requirement

First, Figure 7 illustrates the relationship between the system throughput and the minimum QoS requirement, i.e., the minimum achievable rate threshold of the system Q. The number of relays is set to K=3, resulting in a hop distance of 1.25 m. The minimum QoS requirement varies from 1 bps to 10 bps. The results show that the proposed SPM scheme and the exhaustive search-based optimal scheme achieve identical performance, confirming the optimality of the former. Furthermore, it can be observed that the throughput of the proposed scheme meets and equals the minimum QoS requirement. In contrast, the throughput of the three schemes with fixed PS ratios and fixed source node transmit power remains constant regardless of the minimum QoS requirement. Although the suboptimal schemes with fixed PS ratios of 0.5 and 0.75 exhibit higher throughput than the proposed scheme when Q is low, they clearly fail to guarantee the system’s minimum QoS requirement.

#### 4.2.2. Network Lifetime vs. Minimum QoS Requirement

Finally, we evaluate the capability of the proposed SPM scheme in extending the network lifetime. The network lifetime is defined as the time period from the initial state of the source node until its energy is depleted. The number of relays is set to K=3, with a hop distance of 1.25 m. The initial energy of the source node is set to 1 J. We assume that the frame duration used for data transmission is 10 ms. In the theoretical analysis, the execution time of all schemes is assumed to be negligible.

As shown in Figure 8, the proposed SPM scheme achieves a longer network lifetime compared to the suboptimal schemes with fixed PS ratios. For instance, when Q=1 bps, the network lifetime is $1\times 10^{6}$ s; when Q=10 bps, it is $1.42\times 10^{5}$ s, which are 10 times and 1.42 times that of the three fixed PS ratio schemes, respectively. Although the network lifetime of the proposed SPM scheme decreases as the minimum QoS requirement increases, it simultaneously guarantees the minimum QoS requirement, which the three fixed PS ratio schemes fail to achieve.

### 4.3. Impact of Cascade Fading and Practical Considerations

A legitimate concern for any multi-hop system, including the SWIPT-enabled network considered here, is the impact of cascade fading along the multi-hop link, which can lead to inefficient power transfer and signal degradation. This effect results from the cumulative signal attenuation over each hop. However, the proposed closed-form optimal resource allocation scheme is specifically designed to combat this inefficiency. By optimally determining the PS ratio at each relay based on the instantaneous channel gains $g_{k}$, our scheme dynamically balances the trade-off between harvesting enough energy for retransmission and preserving sufficient signal power for reliable ID. The simulation results validate the effectiveness of this approach. Even under the combined effects of large-scale path loss and small-scale Rician fading—which together model a realistic cascade fading channel—our proposed algorithms achieve superior performance compared to suboptimal schemes, as evidenced by the throughput and power minimization results in Figure 4, Figure 5, Figure 7 and Figure 8. This demonstrates that the optimal allocation of resources is key to mitigating the drawbacks of cascade fading, making SWIPT a viable and efficient solution for the practical applications outlined in Section 1.3.

In summary, both proposed schemes not only achieve optimal performance but also feature extremely fast execution speeds, making them highly suitable for practical deployment in WSNs. Moreover, the proposed SPM scheme aligns with the concept of green communication by effectively prolonging the network lifetime.

## 5. Conclusions

This paper focuses on an SWIPT DF multi-hop relaying system applied in WSNs. In this system, relay nodes are equipped with a single antenna and utilize SWIPT technology, enabling battery-free and external power-free operation. This allows passive communication between source and destination nodes, eliminating the manpower costs associated with battery replacement maintenance. It is suitable for applications such as industrial monitoring, remote areas, and emergency communications.

In this work, we investigate two optimization problems: the first aims to maximize system throughput under a given source node transmit power; the second seeks to determine the minimum source node transmit power required to meet a given minimum QoS requirement. We formulate both problems as convex optimization problems and derive closed-form optimal solutions—specifically, the PS ratio combination for the first problem, and the joint PS ratio combination and the source node transmit power for the second problem.

In the simulation section, we compare the two proposed schemes based on the optimal solutions with the exhaustive search-based optimal scheme and suboptimal schemes using fixed PS ratios. The results demonstrate that the two proposed schemes achieve optimal performance while offering extremely fast computational speed, making them suitable for deployment in practical communication systems within the defined application scenarios. While cascade fading presents a general challenge for multi-hop networks, the proposed optimal resource allocation strategy provides an effective means to maintain high efficiency in end-to-end information and power transfer. Furthermore, we highlight the potential of the second proposed scheme in extending network lifetime.

This study is limited to PS-based SWIPT employing single-antenna DF relays. Future work may explore several promising directions to build upon the findings of this paper. First, the resource allocation algorithms should be investigated under practical non-linear EH models that account for saturation and sensitivity effects to provide more accurate performance estimates for real-world deployments. Second, extending the proposed framework to multi-antenna (MIMO) relay nodes represents a significant avenue for enhancing performance; this would involve designing novel beamforming and resource allocation algorithms to jointly optimize EH and ID in the spatial domain, which is expected to yield significant performance gains. Third, research into alternative relaying strategies within the considered multi-hop system, such as AF or TS-based SWIPT protocols, could provide valuable comparisons and extend the applicability of the proposed optimization framework. Fourth, investigating the impact of imperfect CSI and developing robust algorithms for dynamic resource allocation in time-varying channels are also important practical extensions to enhance the resilience and adaptability of the system. Fifth, extending the proposed framework to account for correlated channel conditions is essential for enhancing realism. Future work could integrate spatial correlation models (e.g., using Kronecker or exponential correlation matrices) into the optimization problem and develop robust algorithms to mitigate performance degradation under correlated fading. This would improve the applicability of SWIPT multi-hop networks in industrial IoT scenarios where channel correlations are common.

Finally, beyond the aforementioned model and protocol extensions, the robustness of multi-hop SWIPT networks in real-world deployments must be examined. Key practical aspects include (1) synchronization issues across multiple hops, such as timing, phase, and carrier frequency offsets, which can degrade the coherence of both energy and information transfer, and (2) hardware impairments at transceivers (e.g., power amplifier non-linearities, I/Q imbalance, and phase noise), which are prevalent in low-cost IoT devices and can severely impact the efficiency of energy harvesting and information decoding. Developing resource allocation strategies that are resilient to such practical imperfections would be a vital step toward bridging the gap between theoretical models and real-world applications.

## Figures and Tables

**Table 1:** Comparison of related works on resource allocation for SWIPT relay networks.

	[12,13,16,18]	[19,20,23]	This Work
**Network Type**	Dual-hop	Multi-hop	Multi-hop
**Relay Protocol**	AF/DF	AF/DF	DF
**Relay Power Source**	Battery-assisted EH/SWIPT	Battery-assisted EH/SWIPT/Power beacon	SWIPT-only, battery-free
**Optimization Objective**	Sum rate, Outage, Energy efficiency	Throughput, Battery energy efficiency	STM & SPM
**Solution Methodology**	Iterative algorithms, Heuristics	Iterative algorithms, Heuristics	Unified Closed-form solution

**Table 2:** List of the main symbols.

Symbol	Definition
$r_{0}$	The source node
K	The number of the relays
$r_{k}$	The k -th relay, k∈{1,…,K}
$r_{K+1}$	The destination node
$h_{k}$	The channel coefficient between $r_{k-1}$ and $r_{k}$, k∈{1,…,K+1}
$\mu_{k}$	The PS ratio at the k$\text{-}\mathrm{th}\;\mathrm{relay},\;\mu_{k}\in \left(0,1\right)$, k∈{1,…,K}
$y_{k}$	The received signal at $r_{k}$, k∈{1,…,K+1}
$p_{k}$	$\mathrm{The}\;\mathrm{transmit}\;\mathrm{power}\;\mathrm{of}\;r_{k}$, k∈{0,…,K}
$s_{k}$	The information symbol from $r_{k}$, $E\left[{\left|s_{k}\right|}^{2}\right]=1$, k∈{0,…,K}
$n_{k}^{a}$	$\mathrm{The}\;\mathrm{AWGN}\;\mathrm{introduced}\;\mathrm{at}\;\mathrm{the}\;\mathrm{antenna}\;\mathrm{of}\;\mathrm{node}\;r_{k}$, k∈{1,…,K+1}
$\delta^{2}$	$\mathrm{The}\;\mathrm{total}\;\mathrm{power}\;\mathrm{of}\;n_{k}^{a}$
$y_{k}^{EH}$	$\mathrm{The}\;\mathrm{signal}\;\mathrm{at}\;\mathrm{relay}\;\mathrm{node}\;r_{k}$ for EH, k∈{1,…,K}
$y_{k}^{ID}$	$\mathrm{The}\;\mathrm{signal}\;\mathrm{at}\;\mathrm{node}\;r_{k}$ for ID, k∈{1,…,K+1}
$n_{k}^{c}$	$\mathrm{The}\;\mathrm{AWGN}\;\mathrm{introduced}\;\mathrm{by}\;\mathrm{the}\;\mathrm{ID}\;\mathrm{circuit}\;\mathrm{of}\;\mathrm{node}\;r_{k}$, k∈{1,…,K+1}
$\sigma^{2}$	$\mathrm{The}\;\mathrm{total}\;\mathrm{power}\;\mathrm{of}\;n_{k}^{c}$
$\eta_{k}$	The rectification efficiency at relay node $r_{k}$$,\;\eta_{k}\in \left(0,1\right]$, k∈{1,…,K}
$g_{k}$	The channel gain between $r_{k-1}$ and $r_{k}$, $g_{k}={\left|h_{k}\right|}^{2}$, k∈{1,…,K+1}
$\gamma_{k}$	$\mathrm{The}\;\mathrm{received}\;\mathrm{SNR}\;\mathrm{at}\;r_{k}$ , k∈{1,…,K+1}
$R_{k}$	The achievable rate of for the k -th hop, k∈{1,…,K+1}
W	The transmission bandwidth
R	The end-to-end throughput
$p_{min}$	$\mathrm{The}\;\mathrm{minimum}\;\mathrm{transmit}\;\mathrm{power}\;\mathrm{of}\;\mathrm{the}\;\mathrm{source}\;\mathrm{node}\;r_{0}$
$p_{max}$	$\mathrm{The}\;\mathrm{maximum}\;\mathrm{transmit}\;\mathrm{power}\;\mathrm{of}\;\mathrm{the}\;\mathrm{source}\;\mathrm{node}\;r_{0}$
Q	The minimum achievable rate threshold of the system

## Data Availability

The data presented in this study are available upon request from the corresponding author.

## Abbreviations

The following abbreviations are used in this manuscript:

IoT	Internet of Things
DF	Decode-and-forward
SWIPT	Simultaneous wireless information and power transfer
PS	Power splitting
QoS	Quality of service
KKT	Karush–Kuhn–Tucker
WSN	Wireless sensor network
FSO	Free space optical
RF	Radio frequency
TS	Time switching
ID	Information decoding
EH	Energy harvesting
OFDM	Orthogonal frequency division multiplexing
CD	Chordal distance
IA	Interference alignment
NOMA	Non-orthogonal multiple access
BS	Base station
OP	Outage probability
NLPA	Nonlinear power amplifier
MIMO	Multi-input multi-output
AF	Amplify-and-forward
D2D	Device-to-device
SPM	Source power minimization
STM	System throughput maximization
CSI	Channel state information
AWGN	Additive white Gaussian noise
SNR	Signal-to-noise ratio
LOS	Line-of-sight
